# Suspended lithium niobate acoustic resonators with Damascene electrodes for radiofrequency filtering

**DOI:** 10.1038/s41378-025-00980-w

**Published:** 2025-07-01

**Authors:** Silvan Stettler, Luis Guillermo Villanueva

**Affiliations:** Advanced Nanoelectromechanical Systems Laboratory, École Polytechnique Fédérale de Lausanne (EPFL), 1015 Lausanne, Switzerland

**Keywords:** Electrical and electronic engineering, NEMS

## Abstract

Data rates and volume for mobile communication are ever-increasing with the growing number of users and connected devices. With the deployment of 5G and 6G on the horizon, wireless communication is advancing to higher frequencies and larger bandwidths enabling higher speeds and throughput. Current micro-acoustic resonator technology, a key component in radiofrequency front-end filters, is struggling to keep pace with these developments. This work presents an acoustic resonator architecture enabling multi-frequency, low-loss, and wideband filtering for the 5G and future 6G bands located above 3 GHz. Thanks to the exceptional performance of these resonators, filters for the 5G n77 and n79 bands are demonstrated, exhibiting fractional bandwidths of 25% and 13%, respectively, with low insertion loss of around 1 dB. With its unique frequency scalability and wideband capabilities, the reported architecture offers a promising option for filtering and multiplexing in future mobile devices.

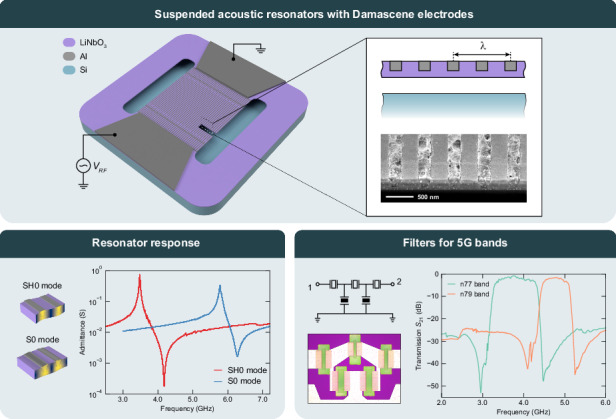

## Introduction

The fifth and sixth generation of wireless communication technology, commonly referred to as 5G and 6G, promise unprecedented advancements in data transfer rates, network capacity, and connectivity. Within the spectrum allocated for 5G new radio (5G NR) deployment, the sub-6 GHz range (5G Frequency Range 1, 5G-FR1) stands out as a crucial spectrum range for large area coverage. The mid-band spectrum (1–6 GHz) strikes an optimal balance between capacity and coverage, making it particularly suited for enabling high-speed mobile services in urban and suburban areas^1^. One of the key factors for enhanced data rates compared to 4G Long Term Evolution (LTE) is the increased bandwidth available with the new 5G NR bands such as the n77 (3.3–4.2 GHz), n78 (3.3–3.8 GHz), and n79 (4.4–5.0 GHz) bands featuring fractional bandwidths (FBW) as high as 24% (n77). The combination of higher frequencies and expanded bandwidths results in increasingly demanding requirements for filter hardware in the RF Front End (RFFE) based on surface acoustic wave (SAW) and bulk acoustic wave (BAW) resonators^2^. At the resonator level, up-scaling the resonance frequency (*f*_*r*_) requires further miniaturization of the acoustic wavelength (λ) and high acoustic velocities (*v*_*p*_), while maintaining low electrical and acoustic losses. Simultaneously, addressing bands with large FBW with acoustic filters necessitates correspondingly high effective electromechanical coupling (*k*^*2*^_*eff*_) of the individual resonator building blocks.

These challenges have motivated advances in incumbent SAW and BAW technologies in the past decade. Even though BAW filter technology has been successful in the 1–8 GHz range^2–6^, the *k*^*2*^_*eff*_ of BAW resonators based on scandium-doped aluminum nitride (AlScN) is not large enough for the broad n77-79 bands. SAW resonators based on lithium niobate (LiNbO_3_) or lithium tantalate (LiTaO_3_) can achieve higher *k*^*2*^_*eff*_ values and *f*_*r*_ can be adjusted by lithography with changes to the electrode pitch of the interdigital transducer (IDT). In recent years, the introduction of multi-layered substrates has enabled substantial progress of SAW technology. The use of substrates consisting of thin films of LiNbO_3_ or LiTaO_3_ bonded to high-velocity materials^7–16^ or Bragg reflector stacks^17^, has improved *k*^*2*^_*eff*_, quality factor (*Q*) and enabled scalability to 3 GHz and beyond.

Resonators exploiting acoustic waves in suspended piezoelectric films such as symmetric^18–22^ and antisymmetric^23–28^ Lamb waves, and shear horizontal (SH) waves^29–33^, have garnered interest as alternatives to classical BAW and SAW resonators. Since the deformation of the piezoelectric layer is not constrained in this configuration, plate waves generally demonstrate higher *k*^*2*^_*eff*_ than what is possible with SAW modes, which facilitates the synthesis of wide bandwidth filters. The suspended structure also reduces leakage of acoustic energy, resulting in low substrate losses even at high frequencies^34^. So far, suspended LiNbO_3_ resonators operating with the first-order antisymmetric Lamb (A1) mode have shown promise for wideband filtering in the 5G mid-band spectrum. However, the achievable *f*_*r*_ in these types of devices is strongly dependent on the thickness of the piezoelectric film. Thus, the lithographic tunability of *f*_*r*_ is limited, which may complicate implementing the required on-wafer frequency shifts for resonators in a filter. Propagating plate waves such as the fundamental shear horizontal (SH0) and symmetric (S0) modes in suspended LiNbO_3_ films provide greater flexibility. Like SAWs, SH0 and S0 modes can be excited with IDTs and thus benefit from lithographically defined *f*_*r*_. Furthermore, *v*_*p*_ of these modes is only weakly dependent on the film thickness, allowing for a wide range of *f*_*r*_ on a single substrate. Although SH0 and S0 resonators with high *k*^*2*^_*eff*_ have been reported^18,29–31,35^ around 1 GHz, scaling to 5G mid-band frequencies has been difficult. Further decreasing the IDT pitch comes with a drastic drop in *k*^*2*^_*eff*_ which would impact filter bandwidth. Reducing the thickness of the LiNbO_3_ film and IDT electrodes can mitigate this tradeoff to a certain extent^30,36,37^, but this presents practical challenges, including mechanical stability issues and increased electrical resistance.

In this paper, we present a suspended LiNbO_3_ acoustic resonator architecture designed to meet the demanding bandwidth requirements of ultra-wide 5G and 6G frequency bands in filters. The key novelty of the proposed resonator is the integration of IDT electrodes that are embedded in the suspended LiNbO_3_ layer using a Damascene process^38–41^ (D-IDT), instead of conventional IDT electrodes deposited on the surface (S-IDT). We show that the D-IDT configuration notably enhances *k*^*2*^_*eff*_ of the SH0 and S0 resonances. The fabricated D-IDT resonators exhibit high *k*^*2*^_*eff*_ in the 1–7 GHz range, reaching 31% at 3.5 GHz (SH0) and 15% at 5.8 GHz (S0) with *Q* on the order of 100. In contrast to other acoustic resonator technologies, the high *k*^*2*^_*eff*_ values allow us to design acoustic filters with FBWs exceeding 20% without compromising out-of-band rejection or relying on external reactive components. Leveraging these SH0/S0 D-IDT resonators, we implement purely acoustic filters covering the full n77 and n79 5G bands, achieving center frequencies of 3810 MHz and 4780 MHz, 3 dB FBWs of 25% and 13%, and insertion losses (IL) as low as 0.8 dB and 1.5 dB, respectively.

## Results

### D-IDT resonator design and concept

**Fig. 1:**
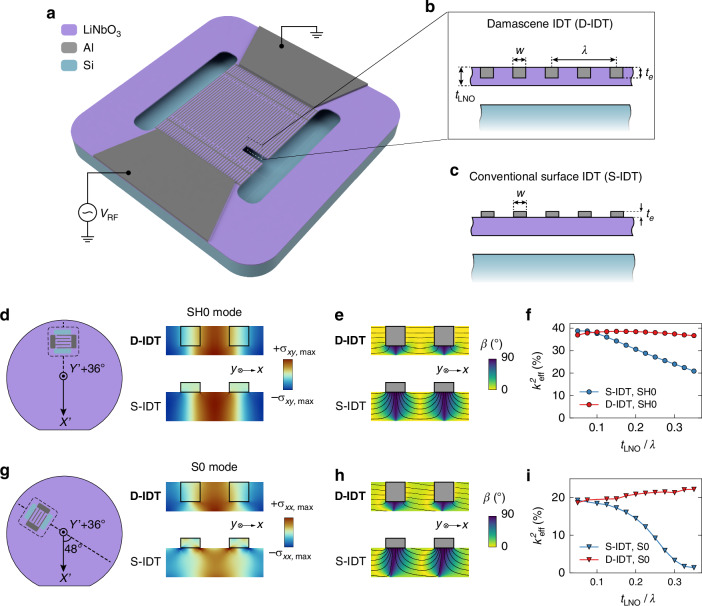
Concept and design of suspended plate resonators with Damascene IDT (D-IDT). **a** Schematic 3D view of a one-port resonator consisting of a suspended lithium niobate film with an array of D-IDT electrodes. **b** Cross-sectional view of the D-IDT configuration compared to (**c**) the conventional surface IDT (S-IDT) configuration. **d**–**f** Comparative analysis of the transduction of the SH0 mode for both transducer configurations. **d** Optimal transducer orientation for SH0 mode excitation with respect to the crystalline X-axis of LiNbO_3_ (denoted X’) and stress distributions. **e** Electrostatic simulations of the electric field applied by the electrodes. **f** Simulated *k*^*2*^_*eff*_ as a function of *t*_*LNO*_/λ. **g**–**i** Comparative analysis of the transduction of the S0 mode for both transducer configurations. **g** Optimal transducer orientation for S0 mode excitation and stress distributions. **h** Electrostatic simulations of the electric field applied by the electrodes. The break in symmetry arises from non-zero off-diagonal values in the permittivity tensor for that orientation of the LiNbO_3_ crystal. **i** Simulated *k*^*2*^_*eff*_ as a function of *t*_*LNO*_/λ. The color in (**e**) and (**h**) shows the angle of the electric field with the horizontal x direction (β)

Figure 1a depicts an overview of the proposed suspended D-IDT resonator with a suspended film of LiNbO_3_ with a periodic array of electrodes with alternating polarity forming the IDT. The IDT is connected to regions with a thick metal layer forming the contact pads. An opening in the LiNbO_3_ film defines the lateral boundaries of the IDT. The free edge parallel to the electrodes acts as an edge reflector confining the acoustic energy in the suspended film. The defining characteristic of a D-IDT resonator is that the aluminum electrodes forming the IDT are embedded in the piezoelectric film (Fig. 1b) rather than deposited on top of it (Fig. 1c). We chose a film of YX36°-cut LiNbO_3_ as the piezoelectric layer. With this specific cut, both the SH0 and S0 modes can be excited with high *k*^*2*^_*eff*_ with an in-plane (parallel to the wafer surface) electric field for specific in-plane orientations of the transducer (see Fig. S1 in the Supplementary Information). Other than the piezoelectric material properties, the *k*^*2*^_*eff*_ depends on how strongly the horizontal component of the exciting electric field overlaps with the stress distribution of the resonating mode. This aspect is highly dependent on the geometry of the transducer. Given that the resonance frequency for the SH0/S0 modes is inversely proportional to λ, scaling to >3 GHz for 5G applications requires a transducer architecture that provides high *k*^*2*^_*eff*_ even when the ratio of film thickness to λ (*t*_*LNO*_/λ) is increased. Figure 1d shows the SH0 mode stress distributions for the D-IDT and S-IDT transducer configurations. Furthermore, we show the in-plane orientation on the wafer of the transducer that maximizes *k*^*2*^_*eff*_ of the SH0 mode. Qualitatively, the distributions of shear horizontal stress appear to be similar for both transducer configurations. In contrast, the electric field distributions arising from an applied voltage at the electrodes are notably different (Fig. 1e). For the S-IDT configuration, the electric field is perfectly horizontal only at the top surface of the film. Under the electrodes, the electric field is primarily vertical and does not contribute to the generation of the intended SH0 wave. With the D-IDT configuration on the other hand, the electric field between the electrodes is horizontal throughout the thickness of the film where the stress is maximum, and areas with vertical components are small. As shown in Fig. 1f, the D-IDT configuration can thus achieve significantly higher *k*^*2*^_*eff*_ for the SH0 mode when *t*_*LNO*_/λ is large. Figures 1g–i show an analogous comparison for the S0 mode. A direction that is offset from the crystalline x axis by 48° maximizes the piezoelectric coefficient that couples to the longitudinal stress field of the S0 mode (Fig. 1g). For the S-IDT configuration, the presence of the electrode on top of the film perturbs the stress distribution which concentrates stress close to the electrodes rather than in the LiNbO_3_. With the D-IDT configuration, the stress maxima are more favorably located in the center of the film where the electric field is uniform and nearly horizontal (Fig. 1h). The simulation data presented in Fig. 1i confirm that the combined effect of the improved stress and electric field distributions in the D-IDT configuration also drastically enhance *k*^*2*^_*eff*_ of the S0 mode. For both modes, the enhancement of *k*^*2*^_*eff*_ is the most pronounced at *t*_*LNO*_/λ > 0.1. This can be explained in part by how the electric field distribution in the S-IDT and D-IDT configurations evolves with *t*_*LNO*_/λ (see Section 2 and Fig. S2 in the Supplementary Information). In Figures S3 and S4, we show simulated admittance responses, *k*^*2*^_*eff*_ and acoustic velocity (*v*_*p*_) of the SH0 and S0 modes with the D-IDT configuration for different thicknesses and sidewall angles of the embedded electrode.

### Device fabrication

**Fig. 2:**
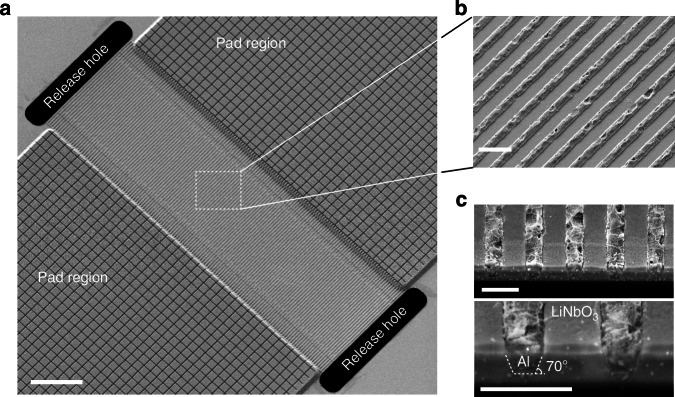
SEM micrographs of a fabricated D-IDT resonator. **a** Overview of a fabricated device with suspended transducer, pad regions, and lateral release holes. Scale bar 10 µm. **b** Close-up view of the D-IDT. Scale bar 500 nm. **c** Cross-sectional views of the transducer obtained by etching a slit through the LiNbO_3_ film. Scale bar 500 nm

Creating a structure with Damascene metal features in the LiNbO_3_ film presents several fabrication challenges, including the patterning of narrow yet sufficiently deep trenches into the LiNbO_3_ film followed by filling with Al without leaving gaps between the metal and sidewalls. We give a detailed description of the fabrication process flow for D-IDT resonators and filters in the “Materials and methods” section. We fabricate our devices on chip level, but the process can easily be scaled to wafer level without any modifications. SEM micrographs showing an overview of a finished device are presented in Fig. 2a. The contact pads, consisting of a grid of embedded metal lines covered with a thick Al layer, are connected monolithically to the D-IDT to ensure maximal electrical conductance. In Fig. 2b, we present a close-up view of the surface of the D-IDT. The polishing process that we developed does not damage the LiNbO_3_ surface between the electrodes. We fabricate resonators with electrode pitches down to 425 nm (λ = 850 nm) while maintaining an electrode width of 220 nm (regardless of λ) for better process uniformity. Figure 2c shows cross-sectional views of the D-IDT highlighting the geometry of the electrodes with a thickness of around 200 nm embedded in the LiNbO_3_ film.

### Resonator characterization

**Fig. 3:**
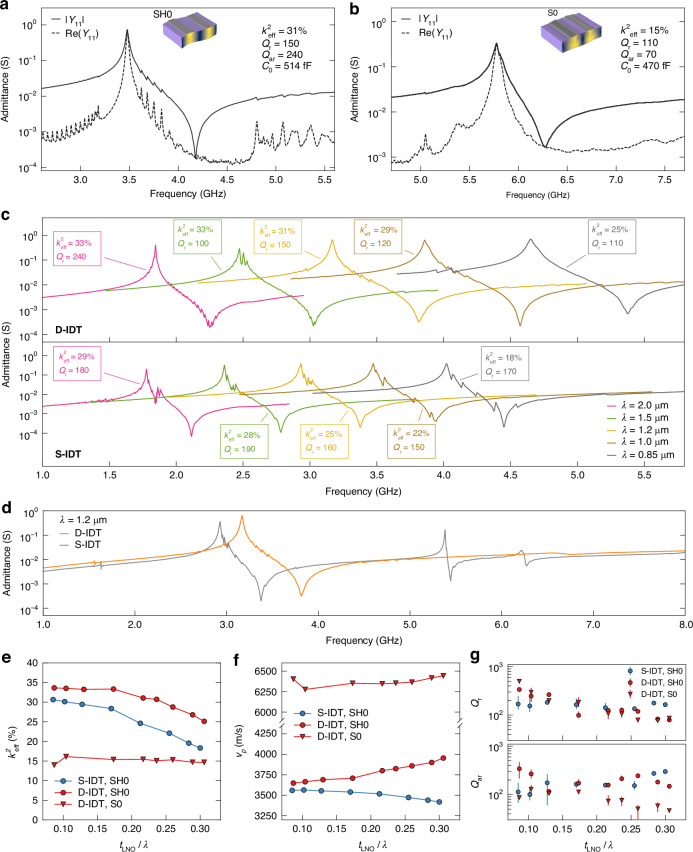
Measured admittance response and comparison of performance metrics of fabricated resonators. Measured admittance of D-IDT resonators with λ = 1.1 μm operating in **a** SH0 mode and **b** S0 mode. The inset mode shapes illustrate the simulated total displacement of the main modes of resonance. **c** Admittance of D-IDT and S-IDT resonators with different λ operating in SH0 mode. For a given λ, the transducer layout for the compared D-IDT and S-IDT resonators is identical. **d** Measured admittance up to 8 GHz of a D-IDT and S-IDT SH0 resonator with λ = 1.2 µm. Summary of extracted **e** *k*^*2*^_*eff*_, **f** *v*_*p*_, and **g** *Q*_*r*_ and *Q*_*ar*_ for D-IDT and S-IDT resonators as a function of the ratio of measured *t*_*LNO*_/λ. The points in (**g**) correspond to the average value of 3 resonators from the same chip, with the error bars representing the minimum and maximum values (covered by the average markers in some cases)

Figure 3a shows the measured admittance of a SH0 D-IDT resonator with a resonance frequency close to 3.5 GHz and in the vicinity of the center of the n77 band. We observe a strong SH0 resonance with *k*^*2*^_*eff*_ over 30% and resonance and anti-resonance quality factors (*Q*_*r*_, *Q*_*ar*_) of over 100 and 200, respectively. Due to the high *k*^*2*^_*eff*_, the response exhibits around 700 MHz of separation between *f*_*r*_ and *f*_*ar*_ with an impedance ratio of over three decades, which is highly promising for large-bandwidth filters. We observe some small ripples between *f*_*r*_ and *f*_*ar*_ which originate from transversal modes. Figure 3b shows the measured admittance of a D-IDT resonator with the same layout as in Fig. 3a but rotated by 48° on the chip surface. Compared to the SH0 mode, the excited S0 mode has lower *k*^*2*^_*eff*_ but higher *v*_*p*_ and thus allows us to attain frequencies higher than 5 GHz with the same λ. Furthermore, the reported performance metrics are reached with static capacitances (*C*_0_) yielding impedances close to 50 ohms, suitable for the implementation of matched filters.

To confirm the superior performance of the D-IDT over the conventional S-IDT configuration, we fabricate a set of SH0 resonators with S-IDT gratings (with 100 nm thick Al electrodes) according to the process flow outlined in ref.^35^. Figure 3c compares measured admittance responses of D-IDT with S-IDT resonators operating in SH0 mode with different *λ*. It is evident that for the same distribution of λ, a broader range of frequencies can be covered with D-IDT resonators due to increased *v*_*p*_ as predicted by simulations (see Fig. S3). At the same time, the drop in *k*^*2*^_*eff*_ is notably smaller, which further confirms their enhanced frequency scaling capability compared to S-IDT resonators. Another critical aspect to consider in the context of an acoustic filter is the presence of spurious modes at frequencies in the desired filter stopband, which may be detrimental to the rejection characteristics of the filter. Figure 3d compares the measured admittance up to 8 GHz of a D-IDT and S-IDT resonator. We note that with the S-IDT configuration, a strong ripple originating from a higher-order plate mode appears close to 5.5 GHz where the WiFi 6/6E bands are located. In contrast, the measured response of the equivalent D-IDT resonator is free of any strong spurious modes, which in a filter ensures high rejection to nearby bands. In Fig. S5 of the Supplementary Information, we present additional D-IDT resonator admittance responses up to 10 GHz and for resonators with λ = 0.9 µm. We also observe that the lithographic alignment of the release holes with respect to the transducer is critical to obtaining a spurious-free response. We provide further information on this issue in Fig. S6 of the Supplementary Information. In Fig. 3e and f, we summarize the extracted *k*^*2*^_*eff*_ and *v*_*p*_ of the measured D-IDT and S-IDT resonators. Remarkably, D-IDT devices operating with the S0 mode exhibit *v*_*p*_ over 6000 m/s and *k*^*2*^_*eff*_ around 15% independent of the chosen acoustic wavelength. Fig. S7 of the Supplementary information shows a comparison of measured and simulated *k*^*2*^_*eff*_ and *v*_*p*_. Figure 3g shows average *Q*_*r*_ and *Q*_*ar*_ values for the fabricated range of *t*_*LNO*_*/*λ. Towards higher *t*_*LNO*_ */* λ ratios, the values of *Q*_*r*_ and *Q*_*ar*_ for D-IDT resonators are decreasing and relatively similar, which suggests that acoustic losses are dominating. While the D-IDT configuration allows for thick electrodes and thus low series resistance, a substantial portion of the resonating body consists of metal with generally much higher intrinsic acoustic losses than monocrystalline LiNbO_3_ (ref. ^34^). For a filter with low IL, the impedance of the series resonators at resonance typically needs to be on the order of 1 ohm or less. Despite the moderate quality factors (~100) above 3 GHz, the D-IDT resonators reach impedances of 1 to 3 ohms at resonance owing to the enhanced *k*^*2*^_*eff*_.

### Wide-band filters for the 5G n77 and n79 bands

**Fig. 4:**
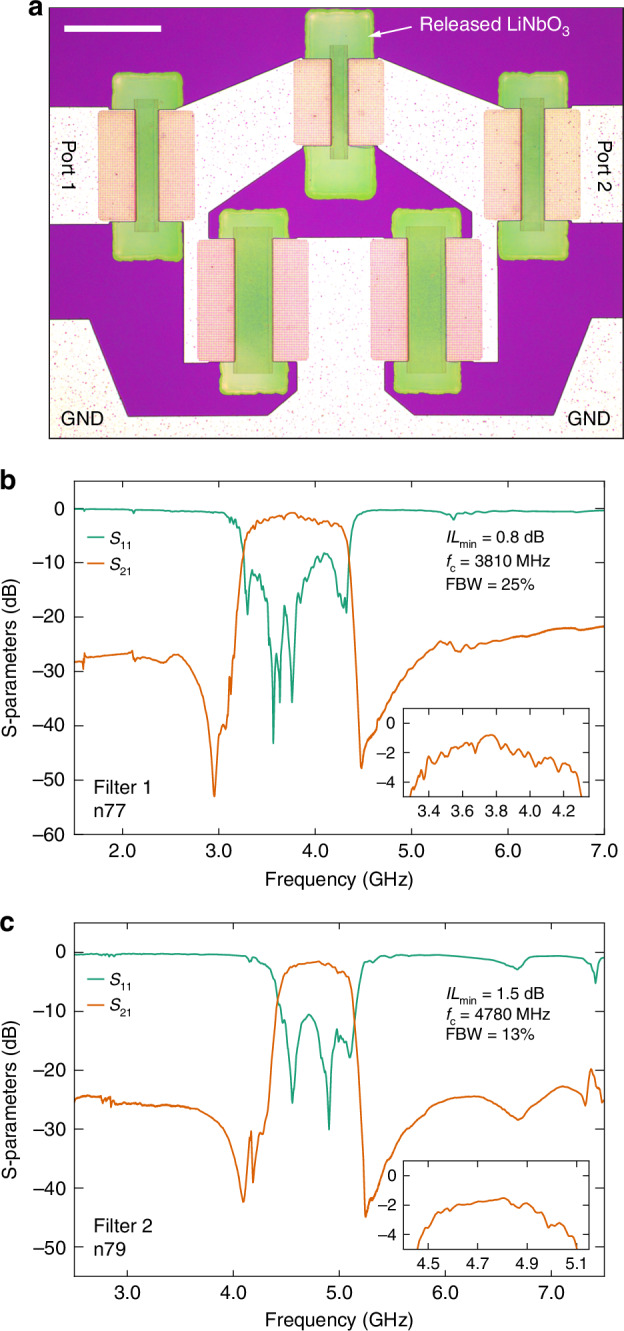
Measurements of filters for the 5G n77 and n79 bands using D-IDT resonator building blocks. **a** Optical microscope image of a fabricated filter with three series and two shunt resonators. Scale bar 100 µm. **b** Measured S-parameters of Filter 1 for the n77 band using D-IDT resonators operating in SH0 mode. The λ of the series and shunt resonators are 1.02 µm and 1.25 µm, respectively. **c** Measured S-parameters of Filter 2 for the n79 band using D-IDT resonators operating in S0 mode. The λ of the series and shunt resonators are 1.30 µm and 1.45 µm, respectively. The macroscopic layouts of Filters 1 and 2 are identical, but Filter 2 is rotated by 48° on the chip surface so that the resonators operate in S0 mode

To demonstrate the suitability of D-IDT resonators for the 5G mid-band spectrum, we synthesize and fabricate ladder-type filters for the n77 and n79 bands. Even though these two bands have notably different center frequencies and FBWs (n77: 3750 MHz, 24%; n79: 4700 MHz, 12.8%), we implement filters for both bands on a single wafer or chip by leveraging the capability to excite two different acoustic modes (SH0 and S0) depending on resonator orientation. For the n77 band filter (Filter 1), we use the high-*k*^*2*^_*eff*_ SH0 mode D-IDT resonators as building blocks to ensure FBW of at least 24% to cover the entire band. For the n79 band filter (Filter 2), we use S0 mode D-IDT resonators, taking advantage of the high *v*_*p*_ of the S0 mode. Figure 4a shows an optical microscope image of a fabricated filter. Since any spurious modes in the vicinity of the main resonances of the resonator building blocks would deteriorate the transmission of the filter, we replace the edge reflectors with grating reflectors for the filter implementation. This design choice does not significantly change *f*_*r*_ or *k*^*2*^_*eff*_ of the resonators, but it avoids any issues related to misalignment which are possible with edge reflectors (see Figures S6 and S8 in the Supplementary Information). To ensure large out-of-band rejection, low IL, and sufficient matching to 50 ohms in the passband, we tune *C*_0_ of the individual resonators by varying the aperture (electrode length) and the number of electrode pairs in the transducer. The measured S-parameter magnitudes of Filter 1 are shown in Fig. 4b. The transmission response features a passband centered at 3810 MHz with an FBW of 25% enabled by the large *k*^*2*^_*eff*_ of the SH0 D-IDT resonators. Thanks to a high *k*^*2*^_*eff*_ ∙ *Q*_*r*_ product, the filter exhibits a small minimum passband IL of only 0.8 dB. Due to the high *k*^*2*^_*eff*_ of the SH0 mode D-IDT resonators, reaching an FBW of 25% does not come at the expense of out-of-band rejection. Filter 1 provides at least 25 dB of rejection in the lower stopband and at least 20 dB up to 7 GHz despite a simple layout containing only five resonators.

Figure 4c shows the response of Filter 2 fabricated on the same chip. The width of the passband corresponds to an FBW of 13%, which is sufficient for the n79 band. The center frequency is slightly higher than required for the n79 band and could be shifted with small adjustments to the acoustic wavelengths of the resonators. The minimum passband IL is higher (1.5 dB) compared to Filter 1 due to the smaller *Q*_*r*_ we obtain above 4 GHz. Filter 2 exhibits similar rejection levels with at least 23 dB in the lower stopband and at least 20 dB up to 7 GHz.

## Discussion

The reported resonator architecture, consisting of a suspended film of LiNbO_3_ in combination with electrodes embedded within the film itself, effectively enables the scalability of SH0 and S0 resonances to the 5G mid-band spectrum. Within that range, the resonator technology we present here offers a unique combination of advantages. Compared to conventional transducer architectures without embedded electrodes, we show that the scaling of *k*^*2*^_*eff*_ towards higher frequencies is notably enhanced with the integration of D-IDT electrodes. In terms of *k*^*2*^_*eff*_, our SH0 resonators outperform their counterparts based on SAWs, which is a substantial advantage for reaching large FBWs in ladder-type impedance element filters. See Table S1 for a comparison of performance metrics with previously published data for other acoustic resonator technologies. In contrast to acoustic resonators that exploit thickness modes in suspended LiNbO_3_ films (A1 Lamb mode, SH1), we can scale *f*_*r*_ of our resonators by simply adjusting the electrode pitch (i.e., λ). Thus, achieving the necessary relative frequency shifts between series and shunt resonators for a ladder-type filter does not require elaborate trimming and can be done on a single substrate.

Unlike technologies with lower *k*^*2*^_*eff*_ such as BAW or SAW, the resonators presented here enable filter FBWs of over 20% without requiring a hybrid design that includes external electromagnetic components or substantial trade-offs in out-of-band rejection^2,42^. With the platform shown in this paper, purely acoustic filters for wide bands can be achieved even with a simple filter design requiring only a handful of resonators and a small footprint. See Table S2, for a comparison of the performance of the filters presented in this work with other acoustic filters in the 3–7 GHz range.

Moving forward, further investigations regarding the compatibility of the presented resonator technology with power handling and temperature stability specifications for 5G and 6G bands are required. Moreover, improvements in resonator *Q* and filter response could be made with further optimization of the fabrication process and a more advanced filter design. Considering future 6G deployment, further increases of *f*_*r*_ up to 10 GHz or more could be envisioned by adopting thinner LiNbO_3_ film and smaller acoustic wavelengths.

## Materials and methods

### Thin film LiNbO_3_ substrates

For device fabrication, we use 4-inch wafers consisting of monocrystalline YX36°-cut LiNbO_3_ thin film with a nominal thickness of 300 nm bonded to a high-resistive silicon wafer purchased from NGK Insulators. The thickness of the LiNbO_3_ layer varies from 255 nm to 315 nm across the wafer. Before device processing, Pt alignment marks are deposited, and the wafers are diced into chips.

### Fabrication process for D-IDT resonators

A schematic outline of the process flow is shown in Fig. S9. The outline of the process is inspired by the widely used Damascene process for interconnects^43^ and previous demonstrations of SAW devices with embedded electrodes^38,39^. The most notable difference in the developed process flow is the absence of Chemical Mechanical Polishing (CMP), which is commonly used but prone to non-uniformities that may render a large portion of devices unusable across the wafer^39,44^. Instead, an alternative polishing process is used that features excellent uniformity, material selectivity, and the possibility of being applied only locally.

### Patterning of the LiNbO_3_ film

To start, a hard mask for LiNbO_3_ etching consisting of a 150 nm Cr layer by e-beam evaporation is deposited. The Cr layer is patterned using e-beam lithography and ion beam etching (IBE, Veeco Nexus IBE350). Next, the underlying LiNbO_3_ film is patterned by Reactive Ion etching (RIE) in an SPTS Advanced Plasma System (SPTS APS) etcher using CHF_3_/Ar chemistry. The etch duration is controlled to achieve a trench depth of 200 nm. The layout of the etched features is designed to avoid aspect-ratio-dependent non-uniformity of the etch rate and ensure that the trench depth is the same for all features. Specifically, all trenches for the electrodes are designed to have a width of 220 nm independent of *λ*. Further, any larger features such as the busbar and pad regions are replaced by dense gratings of trenches with the same width as the trenches for the electrodes. The profile of the etched structures features a flat bottom and a sloped sidewall (~70°). The selectivity of this etch process with the Cr hard mask is roughly 3:1. Thus, around 80 nm of Cr remains on the sample after LiNbO_3_ patterning and serves as a protective etch stop layer at a later stage of the fabrication process.

### Metallization of etched trenches

The etched trenches are filled by evaporating a 10 nm Cr adhesion layer, followed by 600 nm of Al. The sloped trench profile makes it possible to fill in the trenches with evaporation with good coverage of the sidewalls and without any voids in the metal^38^. Due to the directionality of evaporation, the topography of the features in the LiNbO_3_ film remains in the deposited Al film.

### Local planarization with thick photoresist and dry etching

At this point, the thick Al layer covers the entirety of the surface. This step aims to remove the excess Al that is not filling a trench or forming a contact pad. The developed area-selective planarization process consists of planarizing the surface with a thick layer of photoresist (PR), followed by etch-back^45^. After Al deposition, a 700 nm layer of negative tone PR (AZ nLOF 2000, MicroChemicals GmbH, Germany) is spin-coated which fills in topographic features and creates a flat surface on top. The coated PR layer is subjected to a blanket exposure followed by a post-exposure bake to cross-link the entire film. Next, a second 700 nm thick layer of the same PR is spin-coated on top of the first layer and patterned using a maskless aligner (Heidelberg MLA150) to selectively increase the total PR thickness in areas dedicated to contact pads or filter interconnects. The etch-back step starts with IBE until the Cr layer covering the LiNbO_3_ layer between the electrodes is reached. In areas covered with a double layer of PR, the underlying Al layer is not etched during this etch-back process. The protected Al thereby forms monolithic contact pads for the resonator or interconnects for the filters. In areas with only a single PR layer, Al only remains in trenches. However, the resulting surface topography in these areas is not necessarily flat and is dependent on the removal rates with IBE of the materials involved. Interestingly, crosslinked AZ nLOF resist etches slower than Al during the IBE etch-back process, which leads to an inversion of the initial topography and thus excess Al on top of the filled trenches. These Al bumps (~100 nm from the Cr surface) are then flattened with RIE using CHF_3_/Ar chemistry that features a high selectivity to Cr. The Cr layer protects the LiNbO_3_ surface between the electrodes throughout all these etching processes. Finally, the remaining Cr is removed with wet chemistry that does not attack the Al electrodes (TechniEtch Cr01, MicroChemicals GmbH, Germany). Residual PR on the pad regions is stripped using O_2_ plasma.

### Release hole patterning and device release

The lateral release holes are patterned with E-Beam lithography using ZEP520A (Zeon Corp.) E-Beam resist and IBE. After stripping the resist mask, the resonators are released by removing the underlying Si using XeF_2_ gas. Resonators with grating reflectors instead of edge reflectors are released by etching a cavity into Si from the back side of the substrate using Deep Reactive Ion Etching (DRIE) after the planarization step.

### Device characterization and analysis

Individual resonators and filters are measured using a Rhode & Schwarz ZNB-20 Vector Network Analyzer (VNA) with an input power of −15 dBm. The admittance responses of the resonators are fitted with a modified Butterworth-van-Dyke (mBVD) model to extract *k*^*2*^_*eff*_, *Q*_*r*_, *Q*_*ar*_, *C*_0_. *k*^*2*^_*eff*_ is defined as (*f*_*ar*_^2^ *− f*_*r*_^2^)/*f*_*ar*_^2^ according to ref. ^46^.

## Supplementary information

Supplementary information

## Data Availability

The data shown in the plots have been deposited in the Zenodo database https://zenodo.org/records/15578024; 10.5281/zenodo.15578024.
