# Supplementary material for: Suspended lithium niobate acoustic resonators with Damascene electrodes for radiofrequency filtering
Source: Microsyst Nanoeng. 2025 Jul 1;11:131. doi: 10.1038/s41378-025-00980-w (PMC12209459; doi:10.1038/s41378-025-00980-w)
Supplement: Supplementary file 1 — Supplementary information [file 41378_2025_980_MOESM1_ESM.docx]

Supplementary Information:

Suspended lithium niobate acoustic resonators with Damascene electrodes for radiofrequency filtering

Silvan Stettler,^1^* Luis G. Villanueva^1^

^1^ Institute of Mechanical Engineering, École Polytechnique Fédérale de Lausanne (EPFL), 1015 Lausanne, Switzerland

* Corresponding author; [silvan.stettler@epfl.ch](mailto:silvan.stettler@epfl.ch)

# Section 1: Electromechanical coupling and piezoelectric properties of YX36° LiNbO_3_

Fig. S1a illustrates the orientation of the crystalline axis with respect to a reference coordinate system that is aligned with the transducer. In Fig. S1b, we show the piezoelectric coefficients expressed in the reference coordinate system as a function of the in-plane orientation of the transducer. The SH0 mode is excited via the *e_16_* coefficient, which couples horizontal electric field components (along x, index 1 in Voigt notation) to the shear horizontal stress component of the stress field (index 6 in Voigt notation). The S0 mode is primarily excited via the *e_11_* coefficient, which couples horizontal electric field components (along x, index 1 in Voigt notation) to the longitudinal stress component along x of the stress field (index 1 in Voigt notation). It must be noted that the *e_33_* coefficient for YX36°-cut is large, which could excite a longitudinal thickness mode if the electrodes generate any substantial vertical (along z, index 3 in Voigt notation) electric field. Hence, the ideal transducer would only create horizontal electric field components to maximize transduction via *e_11_* and *e_16_* and suppress any longitudinal thickness modes.


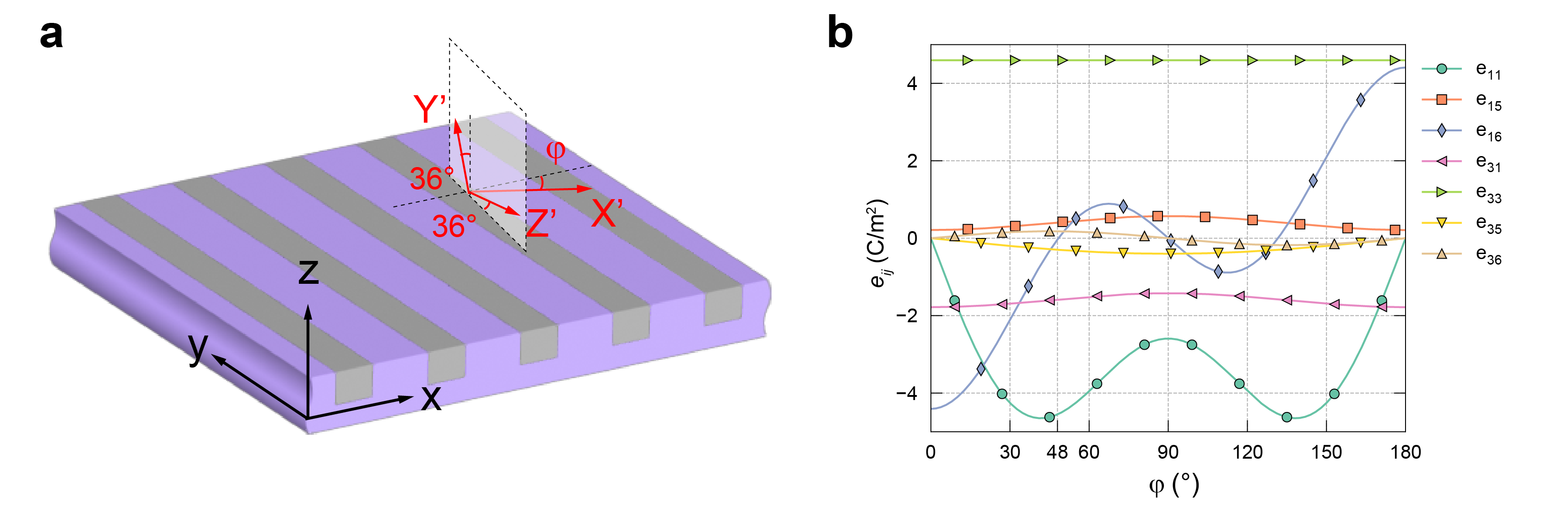


**Fig. S1. Piezoelectric tensor of YX36°-cut LiNbO_3_.** **a** Orientation of the crystalline axis (in red, X’-Y’-Z’) for YX36°-cut LiNbO_3_ relative to a reference coordinate system aligned with the transducer (in black, x-y-z). φ designates the in-plane orientation of the transducer on the chip. **b** Components of the piezoelectric tensor for YX36°-cut LiNbO_3_ expressed in the reference coordinate system for varying φ. The maxima for *e_16_* and *e_11_* are at φ = 0° and close to φ = 48°, respectively. In addition, only one of these two coefficients is non-zero at those orientations, which avoids the excitation of both SH0 and S0 modes simultaneously. Piezoelectric properties for LiNbO_3_ were taken from ref. 1.

# Section 2: Electric field simulations for varying *t_LNO_* / λ

For *t_LNO_* / λ = 0.1, the electrostatic simulations (Fig. S2) show that the electric field is homogenous and approximately horizontal between the electrodes for both transducer configurations. This explains why only a small difference *k^2^_eff_* between transducer configurations can be observed at *t_LNO_* / λ = 0.1 or smaller (Fig. 1f, i in the main text). For *t_LNO_* / λ > 0.1, the fraction of the volume between the electrodes in which the electric field is horizontal decreases for the S-IDT configuration. For the D-IDT configuration, the electric field remains horizontal, independent of *t_LNO_* / λ. This explains why there is an increasing discrepancy of *k^2^_eff_* between the S-IDT and D-IDT configurations towards higher *t_LNO_* / λ (Fig. 1f, i in the main text).


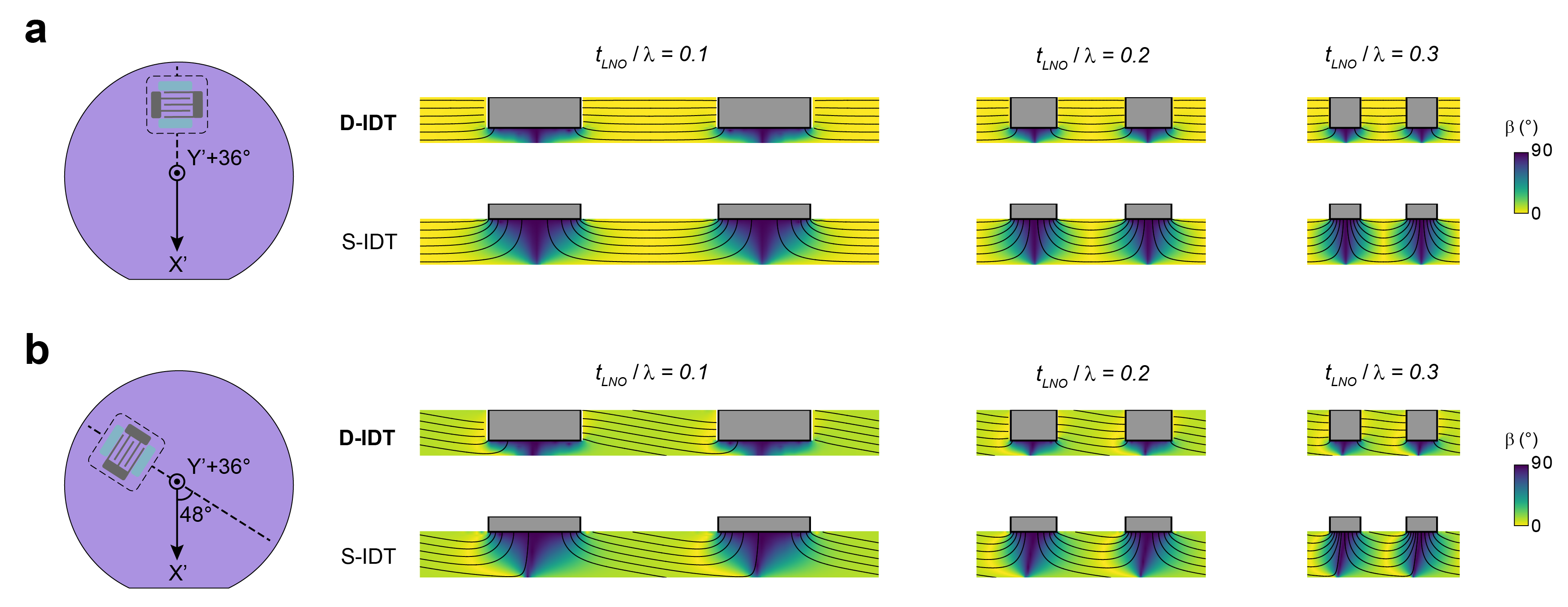


**Fig. S2. Electrostatic simulations of the electric field induced by an applied voltage between the electrodes for varying *t_LNO_* / λ**. Electric field lines and colormaps showing the angle of the electric field with the horizontal direction (β) for the transducer orientations **a** aligned with the crystalline x axis (X’, SH0 mode excitation) and **b** 48° offset from the crystalline x axis (S0 mode excitation). The break in symmetry in **b** arises from non-zero off-diagonal values in the permittivity tensor for that orientation of the LiNbO_3_ crystal.

# Section 3: Simulations of transducer unit cells for varying electrode geometry


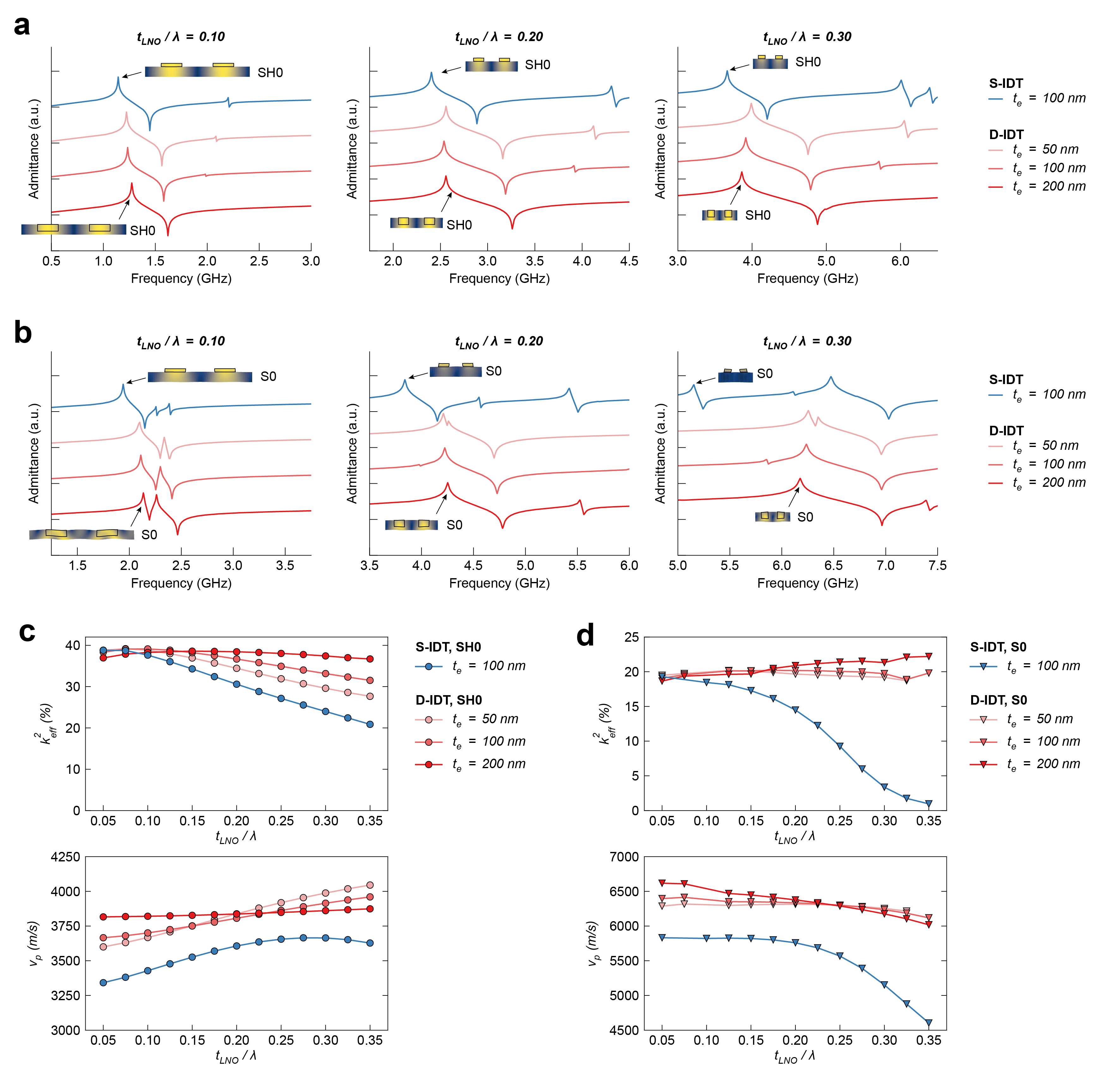


**Fig. S3. Simulated admittance response, *k^2^_eff_*, and *v_p_* for the S-IDT and D-IDT configurations with varying electrode thickness (*t_e_*).** Admittance responses for the **a** SH0 mode and **b** S0 mode. The inset mode shapes show the total displacement of the respective mode. Simulated *k^2^_eff_* and *v_p_* for the **c** SH0 mode and **d** S0 mode. For these simulations, *t_LNO_* = 300 nm and the electrode width is *w* = 0.4∙λ/2.


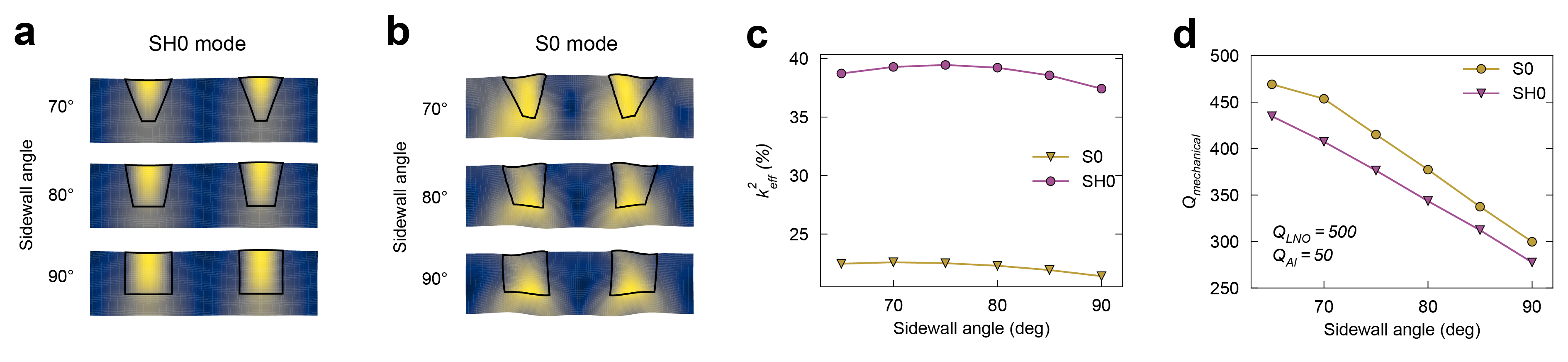


**Fig. S4. Periodic D-IDT unit cell simulations with varying sidewall angle (α).** Simulated mode shapes (color shows total displacement) of the **a** SH0 mode and **b** S0 mode. **c** Dependence of *k^2^_eff_* on the sidewall angle. **d** Extracted dependence of the mechanical quality factor (*Q_mechanical_*) on the sidewall angle. It is assumed that mechanical losses in Al are considerably higher than in LiNbO_3_. Thus, the mechanical losses are set to *Q_LNO_* = 500 and *Q_Al_* = 50, respectively (imaginary part of the stiffness constants). Any other loss sources, such as resistive or dielectric losses, are ignored in these simulations. While *k^2^_eff_* does not significantly change with the sidewall angle, having a lower sidewall angle improves *Q_mechanical_* because the total volume fraction of high-loss Al is reduced simultaneously. For these simulations, *t_LNO_* = 300 nm, *t_e_* = 200 nm, λ = 1 µm, and the electrode width is *w* = 0.4∙λ/2.

# Section 4: Additional measurements


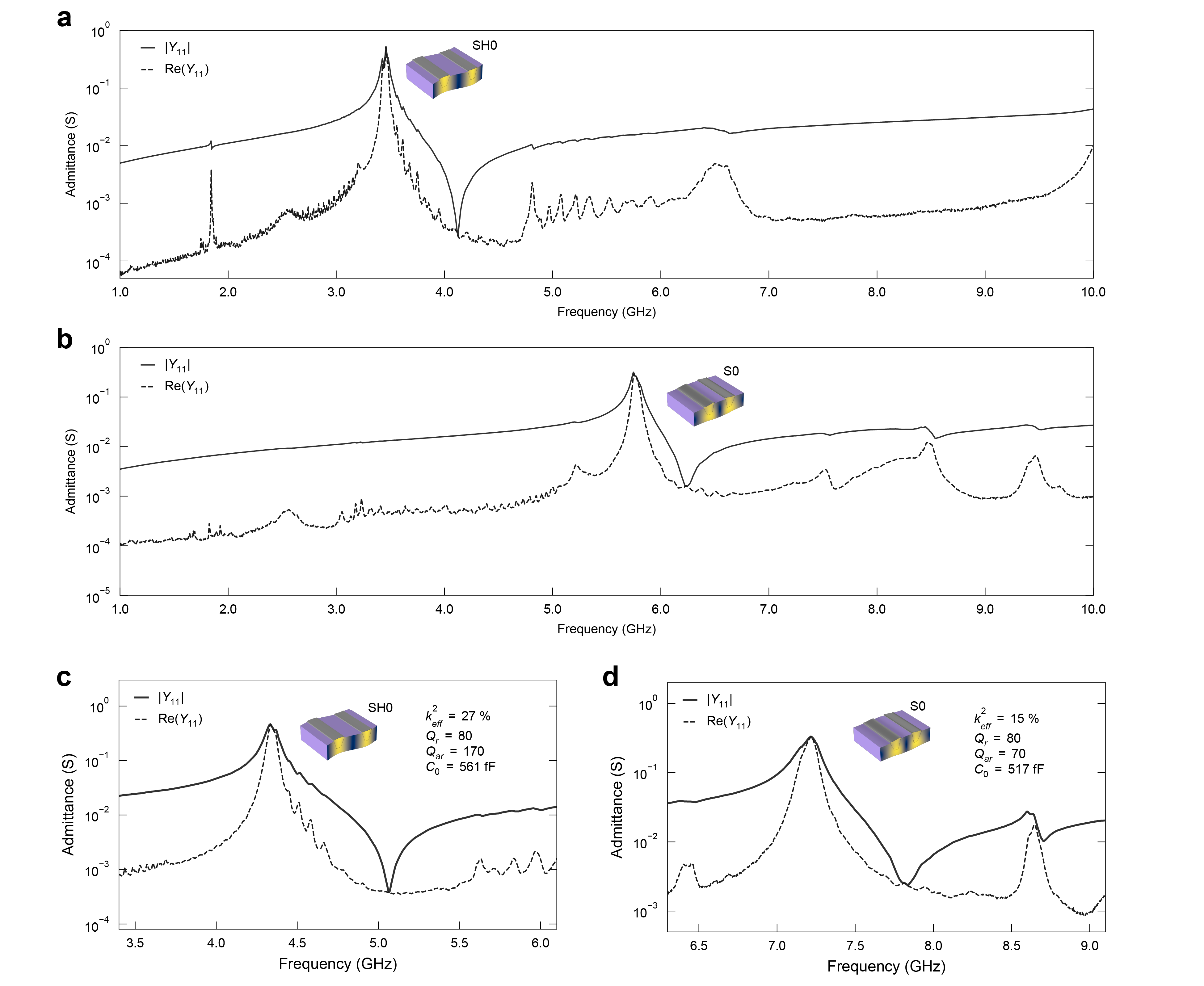


**Fig. S5. Additional measured admittance responses of D-IDT resonators.** Wide-range (1 to 10 GHz) admittance response of D-IDT resonators with λ = 1.1 µm operating in **a** SH0 mode and **b** S0 mode. **c-d** Admittance response of D-IDT resonators with λ = 0.9 µm operating in **c** SH0 mode and **d** S0 mode.

# Section 5: Influence of edge reflector alignment on admittance response


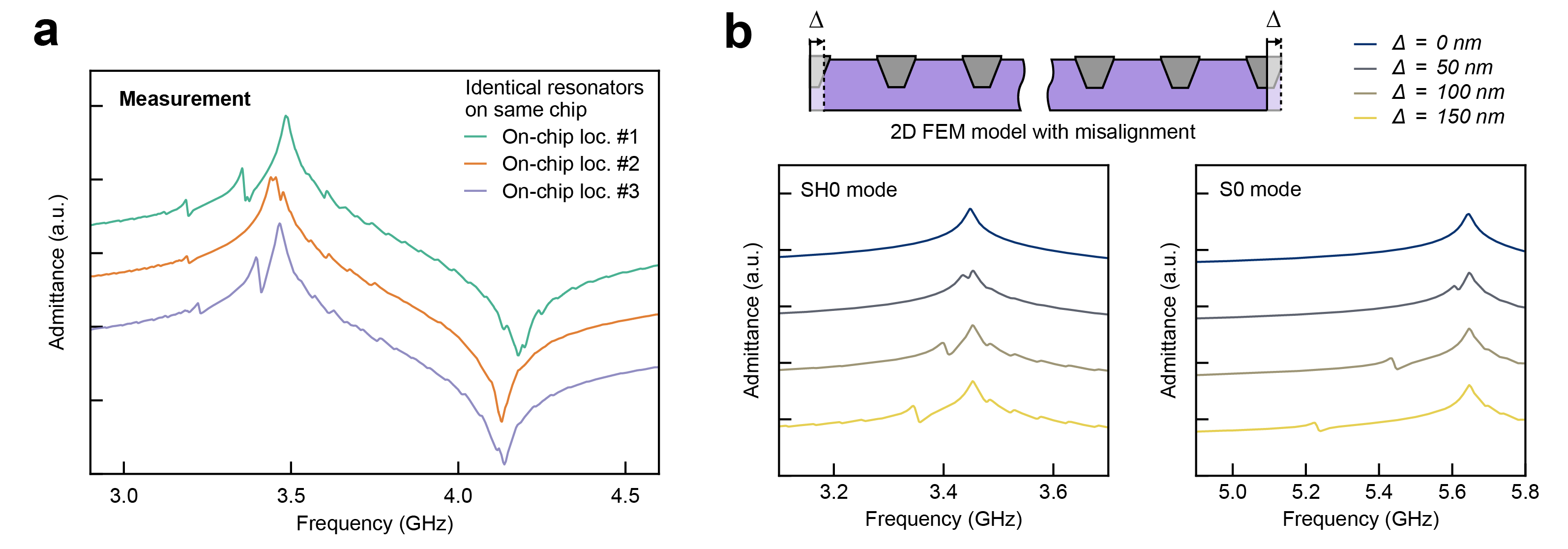


**Fig. S6. Effect of lithographic misalignment of the edge reflectors on admittance response.**
**a** Measured admittance of three identical SH0 D-IDT resonators (with edge reflectors) placed at different locations on the same chip, showing notably varying spurious mode peaks below the main resonance in the response. We observe varying amounts of misalignment up to 150 nm across the chip. **b** Simulated admittance close to resonance for varying amounts of misalignment. For these simulations, we used a 2D finite model that replicates the dimensions of the measured resonators, including the free edges at the D-IDT ends. The position of the free edges is varied relative to their ideal position to mimic lithographic misalignment (Δ). Δ = 0 nm corresponds to edges placed exactly in the center of the last electrodes of the transducer. The simulation results show that a misalignment of 50 nm is sufficient to explain the appearance of the spurious modes around resonance that we observe in some of the measurements.

# Section 6: Comparison of measured and simulated resonator metrics


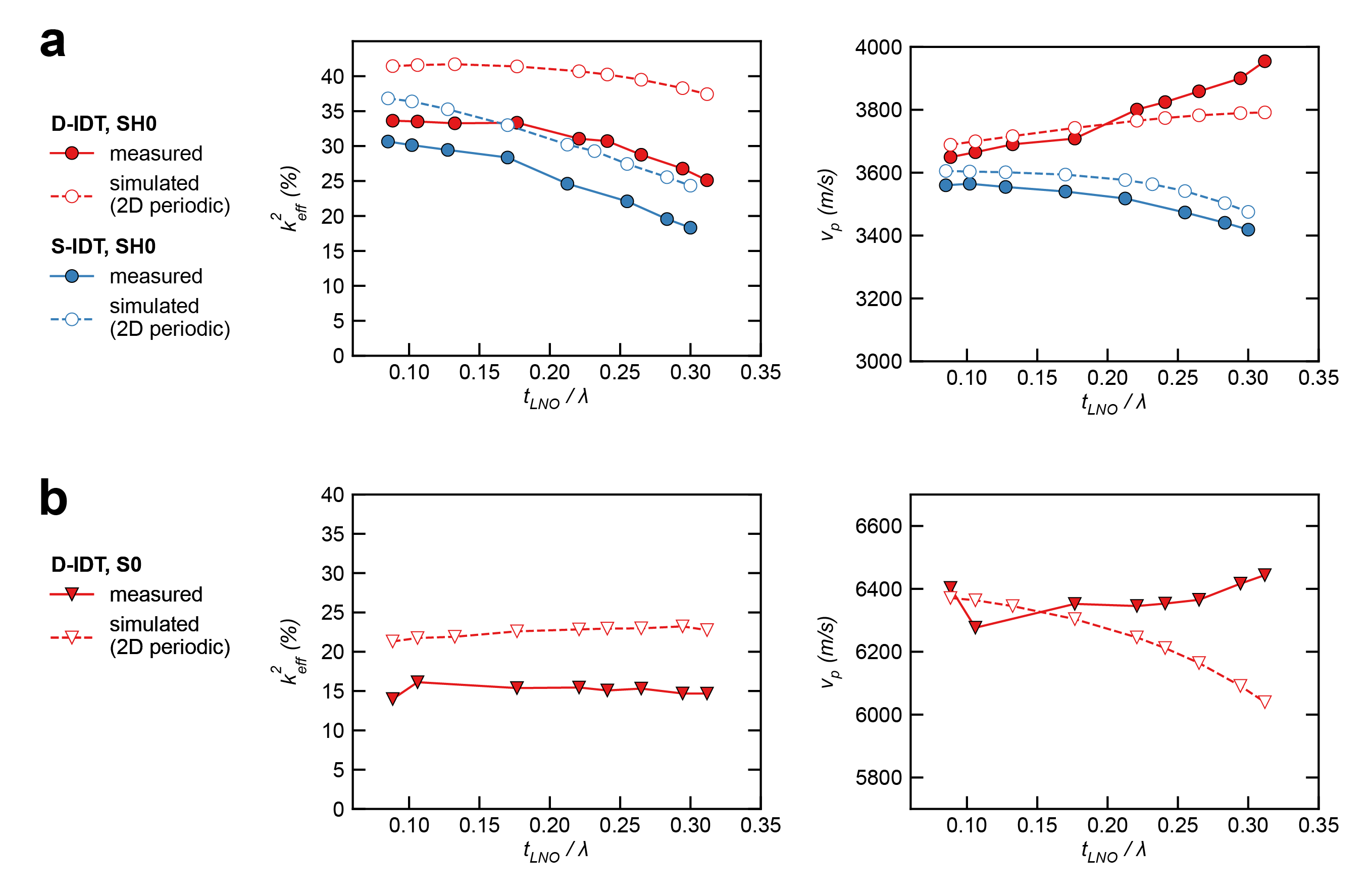


**Fig. S7. Comparison of measured and simulated *k^2^_eff_* and *v_p_*.** Measured and simulated *k^2^_eff_* and *v_p_* for **a** SH0 D-IDT and S-IDT resonators and **b** S0 D-IDT resonators. We use a 2D FEM model of a transducer unit cell with periodic boundary conditions for the simulations. To ensure a meaningful comparison, the parameters of the unit cell are adjusted to the dimensions of the fabricated devices. This includes a finger width *w* = 220 nm independent of wavelength, embedded electrodes with *t_e_* = 200 nm with a sidewall angle of 70°, and measured LiNbO_3_ thickness (*t_LNO_* = 255 nm for the S-IDT chip, *t_LNO_* = 265 nm for the D-IDT chip). Qualitatively, the measured trends agree with the simulations. The periodic 2D model we use for the simulations assumes that the transducer is infinitely periodic. Further, it does not account for parasitic capacitances and the profile of the mode shape along the out-of-plane directions (parallel to the length of the electrodes).

# Section 7: Resonators with grating reflectors


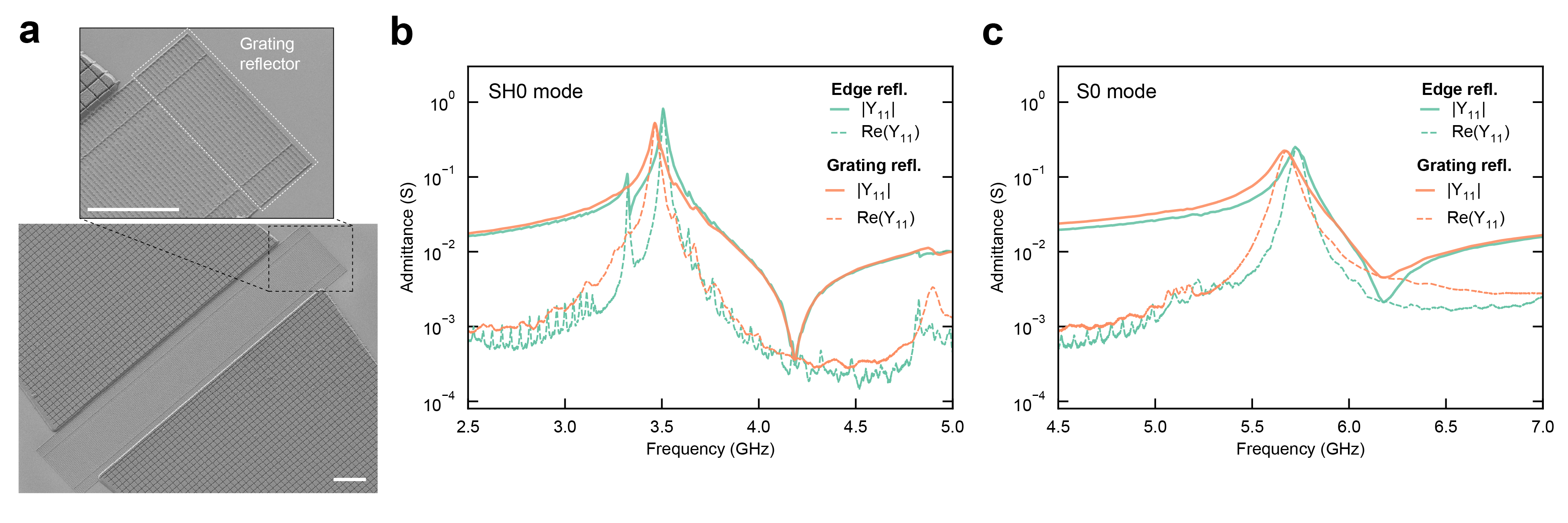


**Fig. S8. D-IDT resonators with grating reflectors. a** SEM micrographs of a fabricated D-IDT resonator with reflectors consisting of a short-circuited grating of Damascene electrodes. Reflector and transducer elements are defined in the same mask which avoids any alignment issues. Scale bar 10 µm. **b-c** Comparison of measured admittance responses of D-IDT resonators (λ = 1.1 µm) with grating reflectors and edge reflectors operating in **b** SH0 mode and operating in **c** S0 mode. The resonance frequencies and *k^2^_eff_* are not significantly different. However, with edge reflectors we observe a spurious mode below the main resonance in **b** due to a slight misalignment of the release holes (Fig. 2 in the main text).

# Section 8: Process flow for D-IDT resonator fabrication


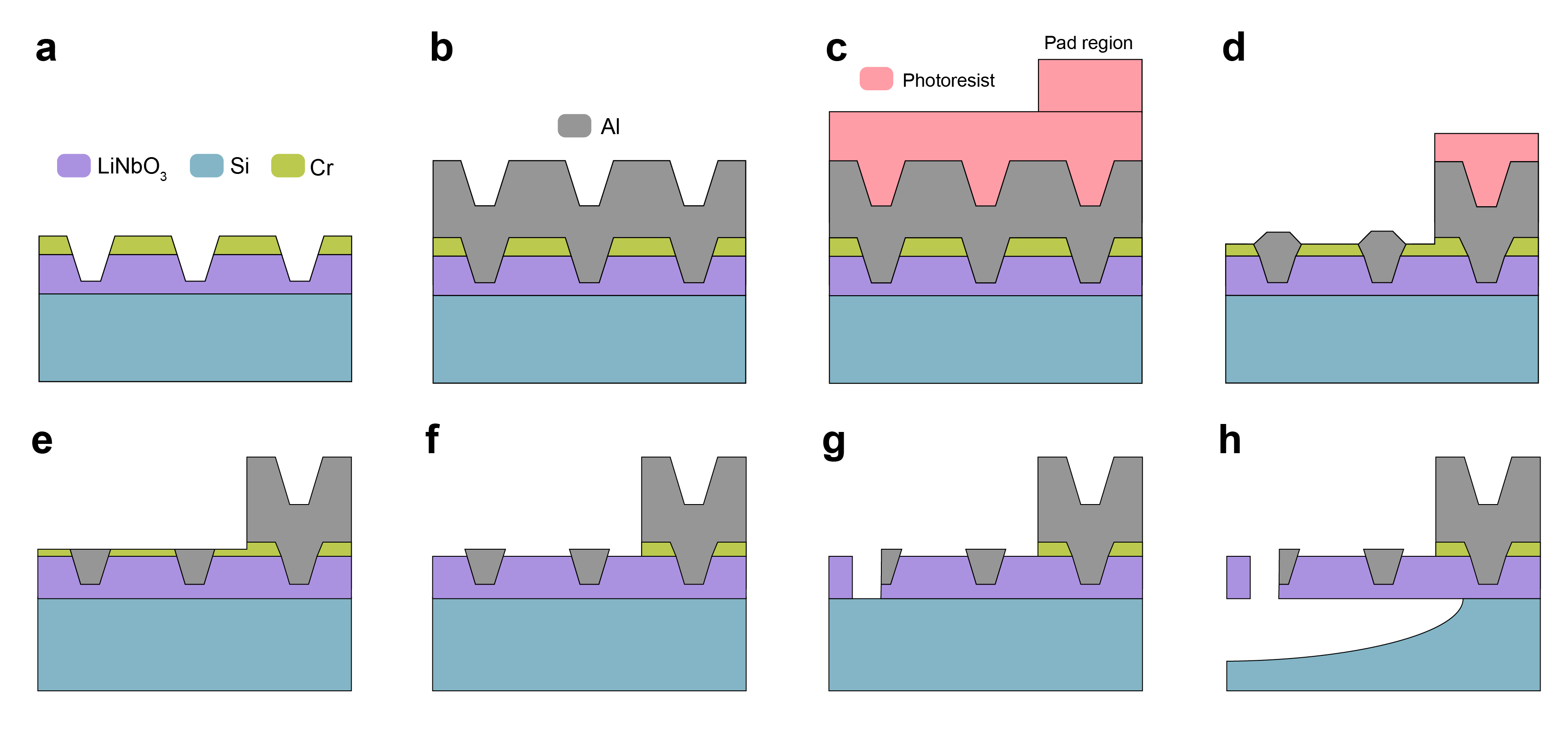


**Fig. S9. Process flow for D-IDT resonator fabrication.** **a** Cr hard mask deposition and patterning, LiNbO_3_ patterning. **b** Al deposition. **c** Photoresist spin-coating to planarize the sample, followed by spin-coating and patterning of a second photoresist layer to define the pad regions. **d** Blanket ion beam etching of the sample until the underlying Cr layer is reached. **e** Reactive ion etching of protruding Al structures. **f** Removal of remaining Cr with wet chemistry. **g** Opening of release holes with e-beam lithography and ion beam etching. **h** Device release with XeF_2_ gas etching.

# Section 9: Comparison with state-of-the-art acoustic resonators and filters

| **Reference** | **Materials** | **Acoustic mode** | ***f_r_* (GHz)** | ***k^2^_eff_* (%)** | ***Q_r_*** | ***Q_ar_*** | ***Q_Bode,max_*** | |
| --- | --- | --- | --- | --- | --- | --- | --- | --- |
| **SAW** | | | | | | | | |
| 2 | X-cut LiNbO_3_/SiC | SH-SAW | 3.6 | 21 | 140* | 500* | | 520 |
| 3 | X-cut LiNbO_3_/SiC | SH-SAW | 3.1 | 18 | - | - | | 350 |
| 4 | YX32°-cut LiNbO_3_/SiO_2_/Si | SH-SAW | 3.8 | 19 | 95 | 1020 | | 1030 |
| 5 | Y-cut LiNbO_3_/SiC | SH-SAW | 5.1 | 16 | 50 | 580 | | 580 |
| 6 | YX32°-cut LiNbO_3_/SiO_2_/SiC | SH-SAW | 3.6 | 20 | 80* | 600* | | 600 |
|  |  | LL-SAW | 5.7 | 9 | 110* | 500* | | 500 |
| 7 | X-cut LiNbO_3_/SiC | LL-SAW | 5 | 14 | 180* | 330* | | 410 |
| 8 | YX42°-cut LiTaO_3_/SiC | SH-SAW | 3.4 | 7 | - | - | | 900 |
|  |  | LL-SAW | 4.9 | 4 | 80* | 1000* | | 1000 |
| 9 | YX32°-cut LiNbO_3_/SiO_2_/Si | LL-SAW | 5.9 | 19 | 90* | 220* | | 220 |
| **Embedded IDT SAW** | | | | | | | | |
| 10 | YX26°-cut LiNbO_3_ | SH-SAW | 3.1 | 27 | < 50* | < 50* | | - |
|  |  | SH-SAW (3^rd^ harm.) | 7.8 | 19 | < 50* | < 50* | | - |
| 11 | YX42°-cut LiTaO_3_/Glass | SH-SAW | 2.5 | 2 | 80* | 60* | |  |
| **Suspended plate wave** | | | | | | | | |
| 12 | X-cut LiNbO_3_ (100 nm, suspend.) | SH0 | 2.9 | 21 | 260 | 210 | | - |
| 13 | X-cut LiNbO_3_ (100 nm, suspend.) | S0 | 6.1 | 14 | 280 | 300 | | - |
| 14 | YX128° LiNbO_3_ (150 nm, suspend.) | S0 | 6.2 | 12 | - | - | | 220 |
| 15 | Z-cut LiNbO_3_ (375 nm, suspend.) | A1 | 5.0 | 20 | 210 | 150 | | - |
| 16 | YX128° LiNbO_3_ (550 nm, suspend.) | A1 | 3.3 | 27 | 390 | - | | - |
| **BAW** | | | | | | | | |
| 17 | AlSc0.3N (400 nm, suspend.) | L-BAW | 4.4 | 18 | 110* | 240* | | 280 |
| 18 | Y-cut LiNbO_3_ (400 nm, suspend.) | S-BAW | 3.7 | 30 | 110 | 230 | | - |
| **This work** | **YX36° LiNbO_3_ (300 nm, suspend.)** | **SH0** | **3.5** | **30** | **150** | **240** | | **-** |
|  |  | **S0** | **5.8** | **15** | **110** | **70** | | **-** |

* Value estimated from *Q_Bode_* data, mBVD circuit parameters, or impedance ratio

**Table S1. Performance comparison of acoustic resonator technologies in the 3 - 7 GHz range.** Values of *k^2^_eff_* are stated using the IEEE definition *k^2^_eff_* = (*f_ar_^2^ - f_r_^2^*) / *f_ar_^2^* (ref. 19).

| **Reference** | **Materials**  **Acoustic mode** | **Center frequency *f_c_* (GHz)** | **Fractional bandwidth (%)** | **Min. insertion loss (dB)** | **Out-of-band rejection**  **(< *f_c_*, > *f_c_*) (dB)** | **# of resonators** |
| --- | --- | --- | --- | --- | --- | --- |
| 2 | X-cut LiNbO_3_/SiC  SH-SAW | 3.76 | 25 | 1.5 | 14, 16 | 8 |
|  |  | 3.56 | 16 | 1.2 | 30, 20 | 11 |
| 4 | YX32°-cut LiNbO_3_/SiO_2_/Si  SH-SAW | 3.73 | 28 | 0.9 | 9, 20 | 9 |
| 20 | YX32°-cut LiNbO_3_/SiO_2_/Si  SH-SAW | 3.62 | 20 | 1.3 | 30, 30 | 9 |
| 7 | X-cut LiNbO_3_/SiC  LL-SAW | 4.84 | 10 | 0.9 | 26, 15 | 5 |
| 21 | X-cut LiNbO_3_/SiC  SH/LL-SAW | 3.95 | 13 | 0.9 | 30, 30 | 5 |
|  |  | 5.70 | 7 | 1.6 | 30, 30 | 5 |
| 22 | Doped AlN  L-BAW (FBAR) | 5.60 | 8 | 2.3 | 50, 50 | 11 |
| 17 | AlSc0.3N  L-BAW (FBAR) | 4.43 | 9 | 2.8 | 10, 10 | 2 |
| 23 | Z-cut LiNbO_3_  A1 | 4.75 | 15 | 1.4 | 30, 35 | 5 |
| 24 | Z-cut LiNbO_3_  A1 | 6.20 | 12 | 1.7 | 10, 10 | 5 |
| 25 | Z-cut LiNbO_3_  A1 | 4.50 | 10 | 1.7 | 15, 13 | 5 |
| **This work** | **YX36°-cut LiNbO_3_**  **SH0** | **3.81** | **25** | **0.8** | **25, 21** | **5** |
|  | **YX36°-cut LiNbO_3_**  **S0** | **4.78** | **13** | **1.5** | **24, 22** | **5** |

**Table S2. Performance comparison of acoustic filters in the 3 - 7 GHz range**

**References**

1. Kovacs, G., Anhorn, M., Engan, H. E., Visintini, G. & Ruppel, C. C. W. Improved material constants for LiNbO3 and LiTaO3. in *Proceedings of the IEEE Symposium on Ultrasonics* 435–438 (IEEE, 2002).

2. Xu, H. *et al.* SAW Filters on LiNbO3/SiC Heterostructure for 5G n77 and n78 Band Applications. *IEEE Trans. Ultrason. Ferroelectr. Freq. Control* **70**, 1157–1169 (2023).

3. Zhang, S. *et al.* Surface Acoustic Wave Devices Using Lithium Niobate on Silicon Carbide. *IEEE Trans. Microw. Theory Tech.* **68**, 3653–3666 (2020).

4. Su, R. *et al.* Over GHz bandwidth SAW filter based on 32° Y-X LN/SiO2/poly-Si/Si heterostructure with multilayer electrode modulation. *Appl. Phys. Lett.* **120**, 253501 (2022).

5. Hsu, T.-H. *et al.* C-Band Lithium Niobate on Silicon Carbide SAW Resonator With Figure-of-Merit of 124 at 6.5 GHz. *J. Microelectromechanical Syst.* 604–609 (2024).

6. Liu, P. *et al.* Monolithic 1–6-GHz Multiband Acoustic Filters Using SH-SAW and LLSAW on LiNbO3/SiO2/SiC Platform. *IEEE Trans. Microw. Theory Tech.* **72**, 5653–5666 (2024).

7. Zheng, P. *et al.* Near 5-GHz Longitudinal Leaky Surface Acoustic Wave Devices on LiNbO3/SiC Substrates. *IEEE Trans. Microw. Theory Tech.* **72**, 1480–1488 (2023).

8. Zhang, L., Wu, J., Zhou, H., Yao, H. & Ou, X. High-Performance Acoustic Wave Devices on LiTaO3/SiC Hetero-Substrates. *IEEE Trans. Microw. Theory Tech.* **71**, 4182–4192 (2023).

9. Liu, P. *et al.* A near spurious-free 6 GHz LLSAW resonator with large electromechanical coupling on X-cut LiNbO3/SiC bilayer substrate. *Appl. Phys. Lett.* **122**, 103502 (2023).

10. Kadota, M., Kojima, T. & Tanaka, S. 2–8 GHz Range High Harmonic SAW Resonator with Grooved Electrodes in LiNbO3. in *Proceedings of the 2021 IEEE International Ultrasonics Symposium (IUS)* (IEEE, Xi’an, China, 2021).

11. Clairet, A. *et al.* Electrode Confined Acoustic Wave (ECAW) devices for Ultra High Band applications. in *Proceedings of the 2023 IEEE International Ultrasonics Symposium (IUS)* (IEEE, Montreal, QC, Canada, 2023).

12. Tetro, R., Colombo, L. & Rinaldi, M. 2–16 GHz Multifrequency X-Cut Lithium Niobate NEMS Resonators on a Single Chip. Preprint at https://arxiv.org/abs/2405.05547 (2024).

13. Tetro, R., Colombo, L., Gubinelli, W., Giribaldi, G. & Rinaldi, M. X-Cut Lithium Niobate S0 Mode Resonators for 5G Applications. in *Proceedings of the 2024 IEEE 37th International Conference on Micro Electro Mechanical Systems (MEMS)* 1102–1105 (IEEE, Austin, TX, USA, 2024).

14. Chulukhadze, V. *et al.* 2 to 16 GHz Fundamental Symmetric Mode Acoustic Resonators in Piezoelectric Thin-Film Lithium Niobate. Preprint at https://arxiv.org/abs/2405.08139 (2024).

15. Yandrapalli, S., Eroglu, S. E. K., Plessky, V., Atakan, H. B. & Villanueva, L. G. Study of Thin Film LiNbO3 Laterally Excited Bulk Acoustic Resonators. *J. Microelectromechanical Syst.* **31**, 217–225 (2022).

16. Lu, R., Yang, Y., Link, S. & Gong, S. A1 Resonators in 128 deg Y-cut Lithium Niobate with Electromechanical Coupling of 46.4%. *J. Microelectromechanical Syst.* **29**, 313–319 (2020).

17. Zhou, C. *et al.* Highly Doped Single Crystal Al1-xScxN Bulk Acoustic Resonators for High-Frequency and Wideband Applications. *IEEE Trans. Electron Devices* **71**, 6329–6335 (2024).

18. Yandrapalli, S. *et al.* Toward Band n78 Shear Bulk Acoustic Resonators Using Crystalline Y-Cut Lithium Niobate Films With Spurious Suppression. *J. Microelectromechanical Syst.* **32**, 327–334 (2023).

19. *176-1987 - IEEE Standard on Piezoelectricity*. (1987).

20. Su, R. *et al.* Scaling Surface Acoustic Wave Filters on LNOI platform for 5G communication. in *Proceedings of the 2022 International Electron Devices Meeting (IEDM)* 4.2.1-4.2.4 (IEEE, San Francisco, CA, USA, 2022).

21. Zheng, P. *et al.* Miniaturized Dual-Mode SAW Filters using 6-inch LiNbO3-on-SiC for 5GNR and WiFi 6. in *2023 International Electron Devices Meeting (IEDM)* 1–4 (IEEE, San Francisco, CA, USA, 2023).

22. Shen, Y., Patel, P., Vetury, R. & Shealy, J. B. 452 MHz Bandwidth, High Rejection 5.6 GHz UNII XBAW Coexistence Filters Using Doped AlN-on-Silicon. in *Proceedings of the 2019 IEEE International Electron Devices Meeting (IEDM)* 17.6.1-17.6.4 (IEEE, San Francisco, CA, USA, 2019).

23. Turner, P. J. *et al.* 5 GHz Band n79 wideband microacoustic filter using thin lithium niobate membrane. *Electron. Lett.* **55**, 942–944 (2019).

24. Wu, Z., Yang, K., Lin, F. & Zuo, C. 6.2 GHz Lithium Niobate MEMS Filter with FBW of 11.8% and IL of 1.7 dB. in *Proceedings of the 2022 IEEE MTT-S International Conference on Microwave Acoustics and Mechanics (IC-MAM)* 98–101 (IEEE, Munich, Germany, 2022).

25. Yang, Y., Lu, R., Gao, L. & Gong, S. A C-band Lithium Niobate MEMS Filter with 10% Fractional Bandwidth for 5G Front-ends. in *Proceedings of the 2019 IEEE International Ultrasonics Symposium (IUS)* 1981–1984 (IEEE, Glasgow, United Kingdom, 2019).
